# Gender inequities in treatment-seeking for sexual and reproductive health amongst adolescents: Findings from a cross-sectional survey in India

**DOI:** 10.1016/j.ssmph.2021.100777

**Published:** 2021-04-02

**Authors:** Sapna Desai, Neelanjana Pandey, Roopal J. Singh, Shikha Bhasin

**Affiliations:** Population Council, New Delhi, India

**Keywords:** Adolescent, Sexual and reproductive health, Treatment-seeking, Gender, Genital infection, India

## Abstract

**Context:**

India's adolescent health policy aims to improve sexual and reproductive health, especially amongst the most vulnerable. There is limited evidence on how gender influences treatment-seeking patterns amongst unmarried adolescents.

**Methods:**

We analyzed data from 11,651 unmarried adolescent boys and girls aged 15–19 from a cross-sectional survey conducted in two large states of India. We conducted sex-disaggregated analyses to estimate the prevalence of symptoms of genital infections and compare treatment-seeking patterns. We identified correlates through multivariable regression and used a conceptual framework to explore structural, household, social and individual factors that influence gender inequities in adolescent sexual and reproductive health.

**Results:**

One in five unmarried adolescents reported symptoms of genital infections, such as burning or discharge, in the past three months. Factors associated with reporting symptoms varied between boys and girls, except for a common correlation with symptoms of depression. At least two-thirds of boys sought treatment, compared to approximately one in four girls (rural: 66.2% boys, 23.1% girls; urban: 69.4% boys, 30.7% girls). Boys primarily sought care from medical shops or private facilities, while girls used both private and government services. Amongst boys, having friends and being in school was associated with seeking treatment (aOR: 11.47; 95% CI: 2.75, 47.87; aOR: 1.95; 95% CI: 1.24, 3.07, respectively). Odds of seeking treatment were higher amongst girls with exposure to any mass media (aOR: 1.93; 95% CI: 1.25, 2.99) and who had discussed puberty with a parent (aOR: 1.98; 95% CI: 1.32, 2.98).

**Conclusion:**

Stark sex differentials in factors associated with symptoms and in treatment-seeking illustrate how structural gender inequities, such as access to economic resources and education, influence sexual and reproductive health amongst adolescents. Along with health system interventions, addressing gender inequities calls for strategies to strengthen parental engagement, social support and girls’ access to resources.

## Highlights

• One in five unmarried adolescent boys and girls in India reported symptoms of genital infections.
• Factors associated with reporting symptoms varied considerably between boys and girls.
• There were wide sex-based differences in seeking treatment: 2 in 3 boys, but only 1 in 4 girls.
• Parental communication, being in school and having friends were associated with seeking treatment.
• Gender-responsive strategies are critical to address underlying inequities that influence health.

## 1 Introduction

The Global Strategy for Women's, Children's and Adolescents' Health calls for increased investment in the health of young people, an area that has not received sufficient attention in most countries (Patton et al., 2016; WHO, 2020). Sexual and reproductive health (SRH) is an area of particular concern during adolescence, due to physical changes as well as the social and gender norms that play a significant role during this period (Patton et al., 2016; Pulerwitz et al., 2019). Common strategies to improve SRH-related knowledge, treatment-seeking and health outcomes amongst adolescents include community-based outreach, peer education, adolescent-friendly health services and school health education (Chandra-Mouli, Lane, & Wong, 2015; Kesterton & de Mello, 2010). Evidence from low and middle-income countries suggests that some interventions have improved SRH-related knowledge and the use of trained providers amongst adolescents, but have had less success in addressing barriers to seeking treatment for reproductive tract and sexually transmitted infections (RTI/STI)(Chandra-Mouli, Lawe-Davies, & Dick, 2010; Newton-Levinson, Leichliter, & Chandra-Mouli, 2016). Acceptability of services remains a persistent challenge for adolescents, especially related to shame, community stigma and provider attitudes regarding sexual activity (Newton-Levinson et al., 2016). Moreover, underlying social determinants, particularly structural inequities and gender norms, play a major role in influencing SRH outcomes amongst adolescents (George, Amin, de Abreu Lopes, & Ravindran, 2020; Malhotra, Amin, & Nanda, 2019; Pulerwitz et al., 2019; Sen, Östlin, & George, 2007).

In India, home to 253 million adolescents, the government introduced a multi-pronged strategy, the *Rashtriya Kishor Swasthya Karyakram* (RKSK), in 2014 to improve adolescent health and well-being (MoHFW, 2014). Achieving equitable and accessible sexual and reproductive health services is a key goal of the program, through adolescent-friendly health clinics, community-based peer educators and clinic-based counsellors. The program, currently implemented nationwide, continues to evolve to address challenges related to human resources and improving adolescents’ awareness of services (Barua, Watson, Plesons, Chandra-Mouli, & Sharma, 2020).

India's National Family Health Survey-4 (2015–16) provides the most recent, nationally representative estimates of symptoms of RTI/STIs and treatment seeking patterns amongst adolescents (IIPS/ICF, 2017). Amongst unmarried adolescents (ages 15–19) who reported engaging in sexual activity, boys and girls reported similar prevalence of genital symptoms such as burning or discharge, ranging between 9 and 15 percent. Among those who reported symptoms, 40.1 percent of boys (95% CI: 31.6, 49.3) sought treatment, compared to 29.5 percent of girls (95% CI: 14.0, 51.8). Similarly, 35.0 percent of married girls (95% CI: 27.8, 43.0) and 20.5 percent of married boys (95% CI: 8.3, 42.6) reported seeking care.

A large body of community-based research in India has consistently identified individual, social, cultural and health system barriers to seeking SRH services amongst women and girls (Sivakami & Rai, 2019, pp. 121–156). Studies indicate that between one-quarter to one-half of married adolescent girls—an especially vulnerable group—seek treatment for reproductive tract infection symptoms (Hussain et al., 2011; Prusty & Unisa, 2013; Sabarwal & Santhya, 2012). Stigma, shame, stigma and lack of appropriate health services emerge as key barriers to seeking care (Barua & Kurz, 2008, pp. 32–46; Prasad et al., 2005). Few community-based studies have estimated the prevalence of infections amongst unmarried adolescents (Kinkor, Padhi, Panigrahi, & Baker, 2019; Sabarwal & Santhya, 2012). Moreover, most community-based research on SRH services in India has focused on girls, with relatively less research on the prevalence of ailments or treatment-seeking patterns amongst boys (Sivakami & Rai, 2019, pp. 121–156). Qualitative research indicates that boys have very limited sources of information on SRH, and that norms regarding gender and masculinity influence both preventive behaviour and treatment-seeking patterns (Char, Saavala, & Kulmala, 2011; Pande et al., 2006; Verma et al., 2006).

Addressing the gender inequities that shape adolescent SRH is critical to achieving equitable health outcomes (George et al., 2020; Sen et al., 2007). Further, given that less than half of adolescents with symptoms of an RTI/STI reported seeking care in the National Family Health Survey-4 (IIPS/ICF, 2017), identifying factors associated with seeking treatment can inform interventions. This paper aims to provide insight into adolescent SRH in India through an analysis of the prevalence of symptoms of, and treatment-seeking patterns for, genital infections amongst boys and girls in two large Indian states. We examine how multiple facets of adolescents' lives—socioeconomic status, education, health awareness, number of friends, and parental relationships—influence sexual and reproductive health amongst unmarried female and male adolescents, as well as explore gender inequities at the individual, social, economic and institutional level (George et al., 2020; Pulerwitz et al., 2019).

## 2 Material and methods

### 2.1 Setting

We analyzed data from a cross-sectional survey of adolescents aged 10–19, conducted in Bihar and Uttar Pradesh, India in 2015–16. The survey was part of the Population Council's UDAYA (Understanding the Lives of Adolescents and Young Adults) study, which aims to describe the situation of adolescents and their transitions to young adulthood, spanning education, health, relationships, skills and employment, political and civic participation. The study population comprised 1 in 4 adolescents in India, or 1 in 16 globally (Census/UNFPA, 2014; Santhya et al., 2017). Both states are largely rural: approximately 11 percent of Bihar's population and 22 percent of Uttar Pradesh resides in urban areas (Chandramouli, 2011). Human development indicators reflect challenges for health and well-being in both states. At least one-third of the population of Bihar and Uttar Pradesh is categorized as below the poverty line. Literacy is high amongst adolescents, at 81 percent in Bihar and 86 percent in Uttar Pradesh. The 2017 NITI Aayog Health Index of India, a composite score of health sector performance, ranked Uttar Pradesh the lowest, followed by Bihar, amongst India's 21 large states (NITIAayog, 2017).

### 2.2 Sample and respondents

The UDAYA survey was conducted in 2015-16 among a state-representative sample of adolescents aged 10–19 years in Uttar Pradesh and Bihar. The survey focused on five categories of respondents: younger adolescent boys and girls (10–14 years); unmarried boys and girls (15–19 years); and married girls (15–19 years). A multi-stage systematic sampling design used the 2011 census listing as the sampling frame to select rural villages and urban wards independently. Sampling domains were stratified by region, village/ward size, proportion of scheduled caste and scheduled tribes, and female literacy. Sample size was calculated using the prevalence of pre-marital sex, the indicator with the lowest-prevalence in the study population, in line with previous studies amongst adolescents in India (IIPS/PopCouncil, 2010). The survey interviewed more than 20,000 adolescents, with an overall response rate of 92%. This paper reports analyses of data collected from 11,651 unmarried adolescent boys and girls aged 15–19. We excluded married girls and adolescents aged 10–14 from this analysis of gender inequities and SRH, as UDAYA did not survey married boys aged 15–19 or ask SRH questions to younger adolescents.

### 2.3 Survey and variables

The UDAYA study aimed to describe multiple dimensions of adolescents’ lives: education and employability; communication, mobility and decision-making; health; violence; media and technology; parental engagement; and government entitlements. The complete list of indicators is described elsewhere (Santhya et al., 2017). Definitions of variables used in this analysis are reported in Appendix Table 1. To estimate prevalence, adolescents were asked if they had experienced any symptoms of genital infection in three months preceding the survey. Symptoms included were genital ulcers, itching in genitals, swelling in the groin, burning while passing urine, white discharge among girls and urethral discharge among boys. Each symptom was asked separately with a binary yes/no response. Respondents who reported yes to experiencing any symptoms were further asked if s/he had sought treatment, and where. Response options for the latter included government and private health facilities, unregistered medical practitioners, traditional healers, frontline workers (ASHA, *anganwadi* worker, auxiliary nurse midwife), medical shops and home remedies.

### 2.4 Ethical considerations

The Population Council Institutional Review Board provided ethical approval for the study. Adolescents provided individual written consent to participate in the study, along with a parent/guardian for adolescents younger than 18. The Population Council identified referral services for counseling and health services to offer respondents if necessary, and fieldworkers were trained on ethical issues and sensitivity. In addition, interviewing boys and girls in separate segments helped minimize issues related to confidentiality and response bias.

### 2.5 Conceptual frameworks on gender inequities and gender norms

Adolescent SRH outcomes are associated with an intersection of individual factors and underlying gender norms (Malhotra et al., 2019). Gender norms, as defined by Cislaghi and Heise (2020), are “social norms defining acceptable and appropriate actions for women and men in a given group or society” (Cislaghi & Heise, 2020). We drew from two recent conceptual frameworks on gender and adolescent SRH to expand our analysis beyond individual risk factors to examine sources of gender inequities in health outcomes at the economic, institutional, individual and social levels. (George et al., 2020; Pulerwitz et al., 2019). George et al. call attention to structural gender inequities—such as economic security, division of labor, political participation and social norms—that ultimately influence individual-level risk factors. Factors linked to individual outcomes include exposure to reproductive and sexual health risks, vulnerability linked to agency and autonomy, access to and control over resources and access to information. Pulerwitz et al. (2019), expanding on the ecological approach, identify four domains—institutional, individual, social and resources—that intersect with prevailing gender and social norms and power dynamics. Both frameworks underscore the importance of examining underlying structural factors that influence gender inequity during adolescence, such as access to education, as well as those specific to SRH, such as awareness of health services.

**Fig. 1:**
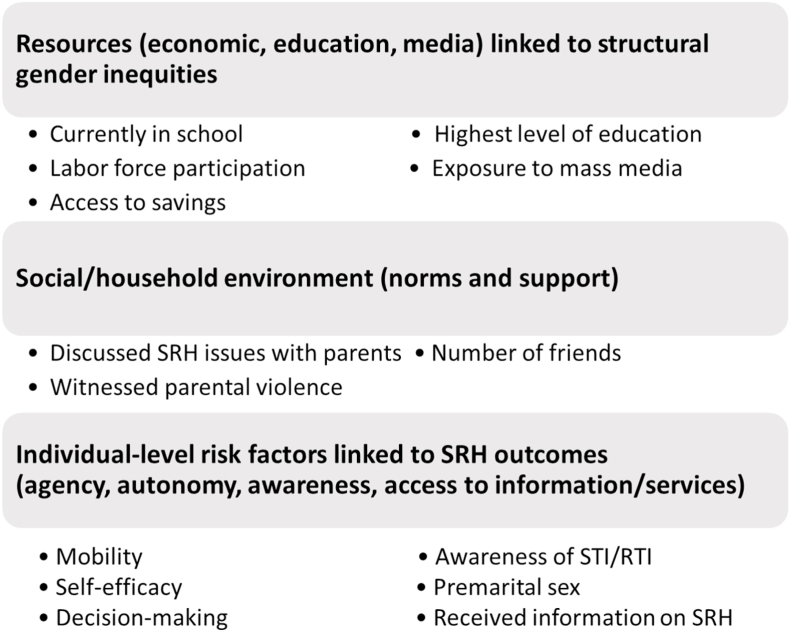
Indicators selected to examine gender inequities and adolescent sexual and reproductive health.

We identified available indicators in the UDAYA survey that reflect individual resources and/or structural inequities, such as currently being enrolled in school, exposure to mass media or access to a savings account, along with individual-level vulnerabilities or risk factors specific to SRH (George et al., 2020). We also included variables that reflect the household and social environment and gender norms (Pulerwitz et al., 2019). Fig. 1 presents the three domains and the corresponding indicators we included in analyses. We also referred to these conceptual frameworks to interpret our findings and identify implications for interventions, particularly in light of the limitations of a cross-sectional survey to examine the complex intersections of gender, economic status and social identity (Bauer, 2014).

### 2.6 Analysis

We present four statistical analyses in this paper: (i) descriptive, sex-disaggregated characteristics of urban and rural unmarried adolescents; (ii) estimates of the prevalence of symptoms of genital infection and description of treatment-seeking patterns, disaggregated by sex and urban/rural residence; (iii) multivariable logistic regression to identify factors associated with reporting a genital infection in the last three months; and (iv) multivariable logistic regression to identify factors associated with seeking treatment, amongst unmarried boys and girls who reported symptoms. We conducted separate analyses for boys and girls, due to the different etiology and symptoms of genital infections. The first two analyses provide weighted estimates with 95% confidence intervals, stratified by sex and rural and urban area.

For the third and fourth analyses, we first used bivariate logistic regression to report unadjusted odds ratios, drawing from the conceptual frameworks above and the literature on RTI/STIs amongst adolescents in India (Kinkor et al., 2019; Nagarkar & Mhaskar, 2015; Sabarwal & Santhya, 2012). We examined several factors in both models: location; state; religion; caste; household wealth index quintile; currently in school; highest level of education attained; mother's education; engaged in paid work in the last 12 months; decision-making; mobility; have any savings; exposure to mass media; peer network; self-efficacy; witnessed domestic violence; discussed SRH issues with parents; received any information on SRH in school/community; awareness of RTI/STIs and history of premarital sex. In addition, we included the Patient Health Questionnaire-(PHQ-9) score for boys and girls to examine associations between reporting depression and symptoms of genital infections (Patel & Oomman, 1999), as well as the use of sanitary napkins for girls (Baker et al., 2017). Factors with evidence of an association in the unadjusted analyses (p ≤ 0.05) were retained in adjusted multivariable logistic regression analyses, controlling for state, location, religion, caste and household wealth quintile. We used the adjusted Wald test to obtain p values for variables with more than two categories. Analyses were performed in Stata 13, using the ‘svy’ command to account for cluster sampling design and survey weights for combined estimates across states. Findings are reported according to the STROBE guidelines for observational studies.

## 3 Results

### 3.1 Household and individual characteristics

Household characteristics (Table 1) indicate that adolescents in urban areas were demographically more diverse, specifically regarding religious and caste identities, compared to rural areas. Adolescents from urban households reported higher levels of household wealth and maternal education than rural areas. Table 2 presents individual characteristics of adolescents, including education, employment, decision-making, exposure to violence and sexual and reproductive health-related information, stratified by sex and rural/urban location. Amongst rural adolescents, boys were generally at an advantage compared to girls across all indicators. In urban areas, school enrolment was similar between boys and girls (approximately 69%), but boys had higher labor force participation, exposure to mass media, mobility, number of friends and access to their own bank accounts. At least four-fifths of adolescents reported frequent exposure to mass media, with the exception of lower exposure amongst rural girls (56.5%). Regarding SRH, two-thirds of girls reported being able to speak with their parents about puberty, compared to less than 10 percent of boys. Awareness of STIs was very low, ranging from 9.6% to 13.0%. A small minority reported ever engaging in premarital sex, with a higher proportion amongst boys (5.7% urban, 10.9% rural) than girls (1.7% urban, 2.8% rural).

**Table 1:** Household characteristics of adolescent respondents, by rural/urban location.

	Urban Households (N = 5706) % (95% CI)	Rural Households (N = 5945) % (95% CI)
Household wealth quintile		
1	3.0 (2.2, 4.0)	12.8 (11.3, 14.5)
2	4.6 (3.6, 5.9)	20.6 (18.8, 22.5)
3	10.8 (9.5, 12.3)	24.3 (22.7, 26.0)
4	28.3 (26.2, 30.4)	24.1 (22.2, 26.1)
5	53.4 (49.6, 57.1)	18.2 (15.8, 20.8)
Religion		
Hindu	67.0(60.6, 72.7)	81.0 (76.3, 85.0)
Muslim	31.9 (26.1, 38.4)	18.6 (14.6, 23.3)
Others	1.1 (0.5, 2.6)	0.4 (0.2, 0.8)
Mother's education		
No education	52.5 (48.5, 56.5)	74.1 (71.8, 76.3)
1–7 years	11.4 (10.1, 12.9)	10.4 (9.3, 11.6)
10 years and above	36.1 (32.0, 40.3)	15.5 (13.7, 17.4)
Caste		
Scheduled Caste	15.7 (13.2, 18.7)	25.3 (22.8, 28.1)
Scheduled Tribe	0.4 (0.2, 0.6)	0.8 (0.5, 1.2)
Other Backwards Class	52.2 (48.5, 55.8)	55.4 (52.1, 58.6)
General	31.7 (28.0, 35.7)	18.6 (15.9, 21.6)

**Table 2:** Characteristics of unmarried adolescent boys and girls, by rural/urban location.

	Urban N = 5706			Rural N = 5945		
	Boys N_b_ = 1960	Girls N_g_ = 3746	p value	Boys N_b_ = 1925	Girls N_g_ = 4020	p value
	% (95% CI)	% (95% CI)		% (95% CI)	% (95% CI)	
Currently enrolled in school	69.8 (65.3, 73.9)	69.0 (65.4, 72.4)	0.660	71.5 (68.4, 74.4)	59.8 (56.2, 63.4)	<0.001
**Educational attainment**						
No education	5.9 (4.0, 8.5)	7.0 (5.5, 8.8)	0.570	3.4 (2.4, 4.8)	7.4 (5.7, 9.6)	<0.001
1–7 years	16.1 (13.3, 19.3)	15.5 (13.4, 17.9)		19.5 (16.9, 22.3)	19.5 (16.9, 22.3)	
8 or more years	78.0 (73.8, 81.8)	77.5 (74.2, 80.5)		77.1 (73.6, 80.2)	73.1 (68.9, 76.9)	
Engaged in paid work in last year	34.2 (30.0, 38.8)	16.7 (14.5, 19.2)	<0.001	35.1 (31.9, 38.5)	22.9 (20.1, 26.0)	<0.001
**Mobility**						
Free to visit two or more places	96.8 (95.4, 97.8)	51.8 (47.4, 56.2)	<0.001	97.0 (96.0, 97.8)	37.3 (34.6, 40.1)	<0.001
**Decision-making**						
Takes decisions independently/jointly	49.3 (45.4, 53.3)	68.5 (65.1, 71.7)	<0.001	58.2 (54.6, 61.8)	77.9 (75.1, 80.6)	<0.001
Operates bank/post office account themselves	88.7 (85.7, 91.1)	76.2 (73.4, 78.8)	<0.001	77.4 (73.1, 81.1)	66.2 (63.3, 69.0)	<0.001
Frequent exposure to mass media	93.7 (91.5, 95.3)	88.6 (86.7, 90.3)	<0.001	84.5 (82.1, 86.6)	56.5 (52.5, 60.4)	<0.001
**Number of friends**						
None	2.3 (1.5, 3.4)	5.7 (4.6, 6.9)	<0.001	3.0 (2.3, 4.0)	5.0 (4.1, 6.0)	<0.05
1-4 friends	54.3 (50.6, 58.0)	69.5 (67.3, 71.6)		64.7 (62.2, 67.1)	67.2 (64.8, 69.5)	
5 or more friends	43.4 (39.8, 47.1)	24.8 (22.7, 27.2)		32.3 (30.0, 34.8)	27.8 (25.5, 30.3)	
Witnessed domestic violence at home	12.9 (10.7, 15.6)	18.7 (16.4, 21.2)	<0.001	19.8 (17.4, 22.5)	25.4 (23.1, 27.8)	<0.05
**Patient Health Questionnaire-9 categories**						
Minimal depression	90.1 (87.9, 92.0)	81.5 (79.2, 83.6)	<0.001	90.2 (88.2, 91.9)	83.2 (81.5, 84.8)	<0.001
Mild depression	7.9 (6.2, 9.9)	12.6 (10.9, 14.4)		8.1 (6.5, 10.0)	12.1 (10.9, 13.4)	
Moderate depression	1.6 (0.9, 2.6)	3.9 (3.0, 4.9)		1.3 (0.9, 2.0)	3.2 (2.6, 3.8)	
Moderately severe depression	0.4 (0.1, 0.9)	1.2 (0.8, 1.7)		0.4 (0.2, 0.8)	1.0 (0.7, 1.4)	
Severe depression	0.1 (0.0, 0.6)	0.9 (0.6, 1.4)		0	0.5 (0.3, 0.9)	
**Self-efficacy**						
Express opinion to family elders and/or if someone says or does something wrong to them	58.6 (54.7, 62.4)	58.4 (54.9, 61.9)	0.940	51.8 (48.3, 55.3)	48.9 (46.1, 51.6)	
**Sexual and reproductive health**						
Have had premarital sex	5.7 (4.4, 7.4)	1.7 (1.2, 2.4)	<0.001	10.9 (9.2, 13.0)	2.8 (2.2, 3.7)	<0.001
Discussed SRH issues with parents	9.6 (7.5, 12.2)	69.0 (65.5, 72.4)	<0.001	6.4 (5.1, 8.1)	67.8 (65.3, 70.3)	<0.001
Use sanitary napkins	-	77.2 (74, 80.1)		-	51.6 (48.4, 54.8)	
Aware of at least one STI symptom	12.4 (10.2, 15.1)	13.0 (11.3, 14.9)	0.700	9.8 (7.9, 12.1)	9.6 (8.3, 11.1)	0.900
Received SRH info at school or community	14.2 (11.8, 17.1)	15.2 (13.3, 17.4)	0.570	15.3 (13.0, 17.9)	20.7 (18.4, 23.3)	<0.001
Genital infection symptoms in last 3 months	21.8 (18.8, 25.2)	21.0 (18.8, 23.3)	0.620	28.4 (25.9, 31.0)	20.0 (18.2, 21.9)	<0.001
Sought treatment for genital infection^a^	69.4 (61.4, 76.4)	30.7 (27.1, 34.6)	<0.001	66.2 (61.5, 70.7)	23.1 (19.5, 27.2)	<0.001

a Amongst those who reported symptoms.

### 3.2 Symptoms of genital infections

Approximately one in five adolescents reported symptoms of genital infections in the past three months, with a higher proportion amongst rural boys (rural boys: 28.4%; 95% CI: 25.9, 31.0). Amongst boys, multivariable regression indicated strong evidence (p < 0.05) of an association between symptoms of genital infection and factors related to material and social resources. (Table 3). Reporting symptoms was associated with: having engaged in paid work in the past year (aOR = 1.31; 1.00, 1.70); savings (aOR = 1.32; 1.03, 1.69); five or more friends (aOR = 2.34; 1.00, 5.43); discussed SRH issues with parents (aOR = 2.77; 1.84, 4.16); awareness of STIs (aOR = 1.73; 1.18, 2.52); reporting signs of mild or higher depression on the PHQ-9 scale (aOR for moderate depression = 4.12; 1.93, 8.78); and history of sexual activity (aOR = 2.30; 1.64, 3.27). Boys who reported involvement in household decisions had lower odds of reporting symptoms (aOR = 0.68; 0.54, 0.87). A different set of factors emerged as associated with reporting symptoms amongst girls. Multivariable analysis indicated evidence of higher odds of reporting symptoms amongst girls who reported: greater mobility (aOR = 1.27; 1.06, 1.52); having witnessed domestic violence at home (aOR = 1.71; 1.41, 2.07); signs of mild or higher levels of depression on the PHQ scale (moderate depression: aOR = 2.84; 1.97, 4.09), use of sanitary napkins during menstruation (aOR = 1.28; 1.03, 1.59) and history of sexual activity (aOR = 1.85; 1.19, 2.88).

**Table 3:** Factors associated with reporting symptoms of genital infection in the last 3 months among female and male adolescents, multivariable regression, adjusted OR (95% CI).

Variables	Boys (N = 922)		Girls (N = 1517)	
	Adjusted OR (95% CI)	p value	Adjusted OR (95% CI)	p value
**Location**				
Urban	0.63 (0.48, 0.81)	0.001	0.83 (0.67, 1.02)	0.081
Rural	(b)		(b)	
**State**				
Bihar	1.02 (0.80, 1.31)	0.833	0.69 (0.53, 0.89)	0.005
Uttar Pradesh	(b)		(b)	
**Religion**				
Hindu	(b)	0.420	(b)	0.008
Muslim	1.15 (0.81, 1.64)		1.37 (1.08, 1.72)	
**Caste**				
Other Backwards Class	(b)	0.377	(b)	0.707
Scheduled Caste/Scheduled Tribe	1.18 (0.90, 1.54)		1.08 (0.84, 1.39)	
General	1.12 (0.86, 1.46)		0.95 (0.76, 1.21)	
**Household wealth index quintile**				
1	0.77 (0.48, 1.21)	0.141	0.70 (0.50, 0.97)	0.047
2	0.86 (0.59, 1.25)		0.61 (0.43, 0.86)	
3	1.22 (0.89, 1.68)		0.87 (0.64, 1.19)	
4	0.97 (0.74, 1.28)		0.81 (0.62, 1.06)	
5	(b)		(b)	
**Engaged in paid work in last 12 months**				
Yes	1.31 (1.00, 1.70)	0.043	1.21 (0.97, 1.50)	0.080
No	(b)		(b)	
**Decision-making at household level**				
Takes decisions independently/jointly	0.68 (0.54, 0.87)	0.003		
No role in decision-making	(b)			
**Mobility**				
None			(b)	0.009
Two or more			1.27 (1.06, 1.52)	
**Have any savings**				
Yes	1.32 (1.03, 1.69)	0.023		
No	(b)			
**Frequent exposure to mass media**				
Yes	1.35 (0.92, 1.97)	0.114		
No	(b)			
**Number of friends**				
None	(b)			
1 to 4 friends	1.53 (0.64, 3.64)	0.328		
5 or more friends	2.34 (1.00, 5.43)	0.048		
**Ever witnessed domestic violence**				
Yes	1.24 (0.95, 1.62)	0.100	1.71 (1.41, 2.07)	<0.001
No	(b)		(b)	
**Discussed SRH issues with parents**				
Yes	2.77 (1.84, 4.16)	<0.001		
No	(b)			
**Aware of at least one STI**				
Yes	1.73 (1.18, 2.52)	0.005	1.05 (0.79, 1.39)	0.724
No	(b)		(b)	
**Received information on RTI/STIs in school/community**				
Yes			1.18 (0.93, 1.51)	0.158
No			(b)	
**Patient Health Questionnaire -9 categories**				
Minimal depression	(b)	<0.001	(b)	<0.001
Mild depression	2.83 (1.99, 4.03)		2.45 (1.95, 3.07)	
Moderate depression	4.12 (1.93, 8.78)		2.84 (1.97, 4.09)	
Moderately severe depression	2.65 (0.72, 9.80)		2.27 (1.24, 4.12)	
Severe depression	-		3.68 (1.33, 10.17)	
**Use sanitary napkins**				
Yes	-		1.28 (1.03, 1.59)	0.023
No	-		(b)	
**Ever had premarital sex**				
Yes	2.32 (1.64, 3.27)	<0.001	1.85 (1.19, 2.88)	0.006
No	(b)		(b)	

### 3.3 Treatment-seeking for symptoms of genital infections

**Fig. 2:**
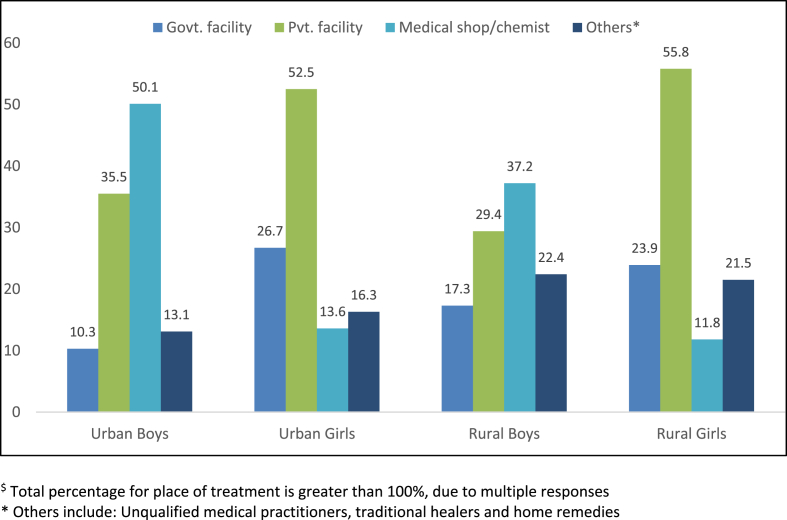
**Place of treatment**^**$**^**for symptoms of genital infection, by gender and rural/urban location (%)**. $ Total percentage for place of treatment is greater than 100%, due to multiple responses. * Others include: Unqualified medical practitioners, traditional healers and home remedies

Amongst adolescents with symptoms, a higher proportion of boys reported seeking treatment than girls: 69.4% boys (95% CI: 61.4, 76.4) vs 30.7% girls (95% CI: 27.1, 34.6) in urban areas, and 66.2% (95% CI: 61.5, 70.7) compared to 23.1% (95% CI: 19.5, 27.2) in rural areas. Boys primarily sought care from medical shops or private facilities, while girls used private facilities, followed by government services (Fig. 2).

Factors associated with treatment-seeking differed between boys and girls (Table 4). Amongst boys, there was strong evidence (p < 0.05) for an association between seeking treatment and having at least one friend (aOR: 11.47, 2.74, 47.90) as well as with currently being enrolled in school (aOR: 1.95; 1.24, 3.07). We found weaker evidence (p < 0.1) for an association between communication with parents on SRH matters and seeing treatment (aOR: 1.96; 0.96, 3.98). Amongst girls, seeking treatment was associated with exposure to any mass media (aOR: 1.93; 1.25, 2.98) and communicating about puberty or pregnancy-related matters with their parents (aOR: 1.98; 1.32, 2.98). Girls who reported having premarital sex had lower odds of seeking treatment (aOR: 0.31; 0.12, 0.78). Household socioeconomic status was not associated with treatment-seeking for girls.

**Table 4:** Factors associated with seeking treatment for symptoms of genital infections, male and female adolescents, multivariable regression (adjusted OR, 95% CI).

Variables	Boys (N = 611)		Girls (N = 397)	
	Adjusted OR (95% CI)	p value	Adjusted OR (95% CI)	p value
**Location**				
Rural	(b)		(b)	
Urban	1.26 (0.78, 2.05)	0.335	1.06 (0.75, 1.49)	0.735
**State**				
Bihar	0.77 (0.5, 1.19)	0.253	0.76 (0.50, 1.16)	0.218
Uttar Pradesh	(b)		(b)	
**Religion**				
Hindu	(b)		(b)	
Muslim	0.92 (0.51, 1.64)	0.782	1.34 (0.88, 2.03)	0.163
**Caste**				
Other Backwards Class	(b)	0.251	(b)	0.064
Scheduled Caste/Scheduled Tribe	0.75 (0.50, 1.12)		0.59 (0.37, 0.94)	
General	0.67 (0.37, 1.20)		0.77 (0.51, 1.16)	
**Household wealth index quintile**				
1	1.07 (0.48, 2.39)	0.276	0.69 (0.33, 1.47)	0.864
2	1.59 (0.85, 2.97)		1.09 (0.47, 2.49)	
3	1.10 (0.57, 2.12)		1.02 (0.53, 1.95)	
4	1.47 (0.82, 2.65)		0.99 (0.58, 1.71)	
5	(b)		(b)	
**Currently in school**				
Yes	1.95 (1.24, 3.07)			
No	(b)	0.004		
**Decision-making at household level**				
No role in decision making			(b)	
Takes decisions independently/jointly			1.38 (0.90, 2.11)	0.135
**Frequent exposure to mass media**				
Yes			1.93 (1.25, 2.98)	
No			(b)	0.003
**Number of friends**				
None	(b)	<0.001		
1 to 4 friends	11.47 (2.74, 47.90)			
5 or more friends	7.49 (1.80, 31.15)			
**Ever witnessed domestic violence**				
Yes			0.78 (0.53, 1.15)	
No			(b)	0.219
**Discussed SRH issues with parents**				
Yes	1.96 (0.96, 3.98)		1.98 (1.32, 2.98)	
No	(b)	0.061	(b)	0.001
**Aware of at least one STI**				
Yes			1.30 (0.82, 2.07)	
No			(b)	0.248
**Ever had premarital sex**				
Yes			0.31 (0.12, 0.78)	
No			(b)	0.014

## 4 Discussion

At least one in five unmarried adolescents in Bihar and Uttar Pradesh reported experiencing symptoms of genital infections in the three months preceding the survey. For boys, reporting symptoms was correlated with factors specific to SRH, such as speaking to parents about SRH, being aware of STIs and having engaged in sex. Girls who reported greater mobility outside the home, witnessing domestic violence within the home, using sanitary napkins and having engaged in sex had higher odds of reporting symptoms. Displaying symptoms of mild, moderate and severe depression was associated with symptoms of genital infections amongst both boys and girls.

Despite similar proportions of boys and girls reporting infection symptoms, there were wide sex differentials in treatment-seeking: 25 percent of unmarried girls reported seeking treatment for symptoms, compared to 67 percent of boys. Boys primarily sought treatment from medical shops and private facilities, while girls preferred private facilities, followed by government services. For girls, access to information—specifically discussion with parents and exposure to mass media—was associated with higher odds of seeking treatment. For boys, having more than one friend and being currently in school—and to a lesser extent, being able to communicate with parents about puberty—was correlated with using services.

### 4.1 Reported symptoms of genital infection

It is unclear why the proportion of unmarried adolescents who reported symptoms of genital infections was slightly higher (approximately 7–10%) than similar surveys amongst unmarried young women (Kinkor et al., 2019; Sabarwal & Santhya, 2012). Self-reports of genital symptoms vary across settings in India, and are generally poorly correlated with clinical diagnosis (Kaida et al., 2018; Kerubo et al., 2016; Koenig, Jejeebhoy, Singh, & Sridhar, 1998). For example, although one-fifth of unmarried adolescent girls (15–19 years) in Kerala reported symptoms of abnormal discharge, only 9.8% of girls had clinically confirmed infections (Nair et al., 2013). Research amongst men has found high prevalence of reported discharge (*dhat)* but low prevalence of clinically confirmed infections (Gautham et al., 2008). Yet even with poor sensitivity, perceived morbidity provides insight into attitudes towards health, and the influence of broader social factors that shape health outcomes and treatment-seeking (Kielmann & Bentley, 2003).

Although sexual activity was correlated with reporting symptoms, the disparity between lower prevalence of previous sexual activity relative to reporting symptoms of genital infections deserves reflection. Reporting bias may be a factor: while the proportion of adolescents who reported sexual activity was very similar to National Family Health Survey-4 estimates in Uttar Pradesh and Bihar, it was lower (especially amongst boys) than findings from an in-depth, mixed-methods study with investigators specifically trained to explore adolescent sexual activity in Maharashtra (Alexander, Garda, Kanade, Jejeebhoy, & Ganatra, 2007). Evidence also points to genital infections not linked to sexual activity. For example, a survey in Odisha reported higher odds of reporting genital infections amongst women who had limited access to toilets (Baker et al., 2017). Our findings indicated an association between reporting symptoms and use of sanitary napkins amongst girls, but no correlation with wealth quintile (which included toilets). These patterns highlight the need to understand the burden of infections not linked to sexual activity, particularly sanitation and hygiene (Anand, Singh, & Unisa, 2015; Baker et al., 2017).

Our findings resonate with previous, albeit limited, evidence on associations between symptoms of genital infection and witnessing domestic violence amongst girls, and with labor force participation amongst boys (Alexander et al., 2007; Sabarwal & Santhya, 2012). While correlations between symptoms of depression and genital infection have been reported amongst adults, the direction of associations and specific mechanisms require deeper understanding, including amongst adolescents (Avasthi, Grover, & Jhirwal, 2012; Gautham et al., 2008; Grover, Avasthi, Gupta, Hazari, & Malhotra, 2016; Jejeebhoy, 2005).

### 4.2 Sex, gender and underlying inequities

Stark sex-based differences in factors associated with reported symptoms and in treatment-seeking reflect structural factors that drive gender disparities and influence health outcomes (George et al., 2020; Malhotra et al., 2019). Our findings point to boys' relative structural and material advantages, with greater opportunities to earn an income, maintain a savings account or go to school. *External* social support—having friends or being in school—was associated with boys' odds of seeking treatment, while girls' use of treatment was associated with factors *inside* the home, specifically communication with parents and exposure to media. For example, boys’ personal access to material resources and mobility outside of the home may provide them the confidence or financial capability to seek treatment. In contrast, girls had greater odds of seeking treatment where parental support on SRH, as reflected by communication on puberty, was available.

Gender norms also govern attitudes towards adolescent sexual activity, such as when unmarried adolescents seek treatment for genital infections (Newton-Levinson et al., 2016). Women's normalization of gynecological symptoms linked to shame is well-documented in India (Puthuchira Ravi & Athimulam Kulasekaran, 2014; Santhya, 2008, p. 252; Sen et al., 2007). However, the small proportion of girls who reported pre-marital sexual activity had lower odds of seeking treatment, but there was no evidence of this association for boys. Despite reporting sexual activity to a surveyor, fear of social sanction may have prevented disclosure to a provider in their own community. Further, the Protection of Children from Sexual Offences (POSCO) Act defines the legal age of consent to be 18 years, which could deter adolescents from seeking treatment from formal providers obligated to report underage sexual activity (Anchan, Janardhana, & Kommu, 2020). Greater use of chemists and the private sector amongst boys, compared to use of both private and government services by girls, may reflects differences in their perceptions of health system access, affordability, acceptability and quality of services (Newton-Levinson et al., 2016; Newton-Levinson, Leichliter, & Chandra-Mouli, 2017).

Although we found no strong evidence that treatment-seeking was independently associated with demographic characteristics such as income, caste or religion, treatment-seeking emerged as associated with having friends and access to information, which may intersect with demographic factors to influence adolescent SRH (Kapilashrami, 2020). Moreover, broader evidence on the utilization of health services in India indicates the importance of recognizing multiple sources of inequity (Baru, Acharya, Acharya, Kumar, & Nagaraj, 2010), and discrimination or vulnerability that may not be captured in a survey.

### 4.3 Implications for interventions

Observed sex differentials in treatment point to the potential of gender-responsive strategies to strengthen health systems for adolescents. Most care sought by adolescents was from private sources, amplifying the need for RKSK to strengthen government services as well as engage the private sector. Specific to boys, forging partnerships with the providers whom they already use, such as medical shops, may improve the quality of care (Collumbien, Mishra, & Blackmore, 2011; Santhya, Prakash, Jejeebhoy, & Singh, 2014). Girls’ low utilization of services may be linked to perceived quality and unwelcoming attitudes amongst providers (Santhya et al., 2014), reinforcing the importance of provider sensitization within RKSK.

Addressing gender inequities in SRH also calls for interventions outside of the health system—particularly capitalizing upon social support and networks in adolescents’ lives (Denno, Hoopes, & Chandra-Mouli, 2015; Jejeebhoy, 2017; Siddiqui, Kataria, Watson, & Chandra-Mouli, 2020; Svanemyr, Amin, Robles, & Greene, 2015). Our findings resonate with previous research on the need to strengthen parental engagement in adolescent programming, especially for girls (Jejeebhoy, 2017; Jejeebhoy & Santhya, 2011). Moreover, working with parents has the potential to influence how gender and social norms are shaped, albeit with variation by family context (Denno et al., 2015; Jejeebhoy, 2017; Malhotra et al., 2019; Pulerwitz et al., 2019).

As evidence builds on effective ways to address gender inequities at multiple levels, research will be required to track, evaluate and adapt program design within and beyond the RKSK (Chandra-Mouli, Lane, & Wong, 2015; Cislaghi & Heise, 2020; Siddiqui et al., 2020). Participatory, group-based activities amongst adolescents, a component of RKSK, hold promise, as do girls’ safe space programs and peer education (Amin, Kågesten, Adebayo, & Chandra-Mouli, 2018; Jejeebhoy, 2017; Rath, Prost, Samal, Pradhan, Copas, Gagrai et al., 2020; Temin & Heck, 2020; Verma et al., 2006). The association between symptoms of depression and genital infections deserves in-depth exploration, as well as how school-based and community-based interventions may improve mental health (Michelson et al., 2020; Rath et al., 2020). Qualitative, context-specific research with adolescents may uncover multiple levels of inequities, and participatory research may identify new opportunities for interventions (Saxena & Yasobant, 2020).

### 4.4 Strengths and limitations

This study analyzed data on SRH amongst unmarried adolescents from a large, population-based survey. Our findings may be limited by reporting biases in sexual history amongst adolescents and self-reported symptoms of genital infections. The survey inquired about treatment-seeking in the past three months, which may be subject to recall bias. Further, this analysis of a cross-sectional survey provided evidence of correlations, but cannot suggest causal factors or determinants of genital infections or treatment-seeking. Lastly, the different aetiology of genital infections in boys and girls prevented analyses of combined data to examine the interactions between gender and other demographic factors.

## 5 Conclusion

It is well-recognised that addresing the social determinants of health and treatment-seeking, including gender norms, should be central to improving health outcomes amongst adolescents (Denno et al., 2015; Haberland, McCarthy, & Brady, 2018; Jejeebhoy, 2017; Malhotra et al., 2019; Svanemyr et al., 2015). Our findings on low treatment-seeking amongst girls highlight the need for the RKSK to invest in community-based and structural interventions that explicitly address gender inequities, alongside ongoing investments in health facility strengthening. Gender inequities that emerge in adolescence will continue to shape access to health care in adulthood, which raises the urgency of addressing adolescent SRH within and beyond the health system.

## Source of Funding

This paper was written using data collected as part of Population Council’s UDAYA study, which is funded by the 10.13039/100000865Bill and Melinda Gates Foundation (Grant # OPP 1111281) and the 10.13039/100000008David and Lucile Packard Foundation (Grant # 2014-40467). No additional funds were received for the preparation of the paper. Funders had no role in the data analysis, interpretation, writing or submission of this article.

## CRediT authorship contribution statement

**Sapna Desai:** Conceptualization, Formal analysis, Writing – original draft. **Neelanjana Pandey:** Methodology, Data curation, Writing – review & editing. **Roopal J. Singh:** Validation, Visualization, Writing – review & editing. **Shikha Bhasin:** Literature review, Writing – review & editing.

## Declaration of competing interest

None.
