# Supplementary material for: Gender inequities in treatment-seeking for sexual and reproductive health amongst adolescents: Findings from a cross-sectional survey in India
Source: SSM Popul Health. 2021 Apr 2;14:100777. doi: 10.1016/j.ssmph.2021.100777 (PMC8095180; doi:10.1016/j.ssmph.2021.100777)
Supplement: Multimedia component 1 [file mmc1.docx]

**Appendix Tables**

| **Appendix table 1: Definitions of indicators used in analyses** | |
| --- | --- |
| **Indicator** | **Definition** |
| Prevalence of genital infections | Adolescents were asked if they had experienced any symptoms of genital infection in three months preceding the survey. Symptoms included were genital ulcers, itching in genitals, swelling in the groin, burning while passing urine, white discharge among girls and urethral discharge among boys. Each symptom was asked separately with a binary yes/no response. |
| Treatment-seeking for genital infections | Respondents who reported yes to experiencing any symptom were further asked if s/he had sought treatment for the infection, and where. Response options for the latter included government and private health facilities, unregistered medical practitioners, traditional healers, frontline workers (ASHA, anganwadi worker, auxiliary nurse midwife), medical shops and home remedies. |
| Decision-making at household level | An index that represents the number of instances where the respondent took a household decision independently or in close conjunction with others among (i) till which standard they will study, (ii) who their friends will be, (iii) mainly takes the decision about making major household purchases, (iv) mainly takes the decision about whether they should work or stay at home. The variable is presented as binary with categories ‘No role in decision making’ and ‘partial/ complete role in decision making’ |
| Mobility | An index constituting the number of places that the respondent was allowed to go alone among (i) friend/relative inside the village/ward, (ii) friend/relative outside the village/ward, (iii) programme (a mela, sports event, girls’ group meetings) inside the village/ward alone. The indicator is presented as binary with categories ‘None’ (permitted to go to none or one of the places alone) and ‘Two or more’ (permitted to go to two or more of the places alone). |
| Frequent exposure to mass media | Is an binary variable that is categorised into: if the respondent was exposed to any form of mass media, almost every day, or, at least once a week among (i) television, (ii) newspaper/ magazine/ books, (iii) radio, (iv) books; and, otherwise. |
| Self-efficacy | Respondents were asked of two instances whether they make a confrontation or not (i) express your opinion to elders in your family, (ii) If someone says or does something wrong to them. Categorised as ‘Express opinion in at least one of the cases’ and ‘Express opinion in neither situation’. |
| Witnessed domestic violence at home | Witnessing domestic violence was measured if they reported that their father ever beat their mother, in a binary response variable. |
| Discussed SRH issues with parents | Respondents were asked if they have discussed in the past year SRH issues with their mother or father such as (i) menstruation (for girls) and (ii) how pregnancy occurs and (iii) physical changes related to puberty. |
| Aware of at least one STI | The respondent was allowed multiple responses on what are the possible symptoms of having an STI. Responses recorded were among: (i) ulcer/sore on private parts, (ii) genital discharge, (iii) itching in private parts, (iv) lower abdominal tenderness/ pain, (v) warts or growths on private parts, (vi) burning or pain on urination, (vii) asymptomatic. |
| Received any service/ information on STIs/ RTIs | Abinary indicator variable that is categorised into: if the respondent received information or services in the last one year from ASHA, AWW or the school doctor or nurse; and, otherwise. Information included: (i) General health and hygiene, (ii) Body change including menstruation and menstrual hygiene, (iii) Nutritious food, (iv) Safe sex practices and STI/HIV/AIDS, (v) Contraception, (vi) Safe pregnancy, (vii) Immunization and infant care, (viii) VHND. Services included: (i) Sanitary napkins, (ii) IFA/De-worming tablets, (iii) Condom/oral pills, (iv) Escorting to health facility, (v) Nutritious food. |
| PHQ-9 categories | Patient Health Questionnnaire-9 is a scale was administered to all respondents to assess the prevalence of depression-related symptoms in the two weeks prior to the interview. This questionnaire is a nine-item depression-screening module; the PHQ-9 score ranges from 0 to 27 with cut-off points of 5, 10, 15, and 20 that represent the thresholds for mild, moderate, moderately severe, and severe depression, respectively (Kroenke, Spitzer, and Williams, 2001). |

| **Appendix table 2: Factors associated with experiencing at least one genital infection in the last 3 months among male and female adolescents, unadjusted OR (95% CI)** | | | | | | |
| --- | --- | --- | --- | --- | --- | --- |
| **Independent Variable** | **Boys  (N=922)** | | | **Girls  (N=1,517)** | | |
|  | **% (n)** | **Unadjusted OR (95% CI)** | **p value** | **% (n)** | **Unadjusted OR (95% CI)** | **p value** |
| **Location** |  |  |  |  |  |  |
| Urban | 21.8% (397) | 0.70 (0.56, 0.88) | 0.003 | 20.9% (742) | 1.06 (0.89, 1.26) | 0.507 |
| Rural | 28.3% (525) | (b) |  | 19.9% (775) | (b) |  |
| **State** |  |  |  |  |  |  |
| Bihar | 25.6% (404) | 0.88 (0.71, 1.10) | 0.287 | 16.6% (574) | 0.72 (0.58, 0.9) | 0.004 |
| Uttar Pradesh | 27.9% (518) | (b) |  | 21.5% (943) | (b) |  |
| **Religion** |  |  |  |  |  |  |
| Hindu | 27.3% (756) | (b) | 0.876 | 19.3% (1028) | (b) | 0.067 |
| Muslim | 26.8% (163) | 0.97 (0.73, 1.29) |  | 22.8% (487) | 1.23 (0.98, 1.53) |  |
| **Caste** |  |  |  |  |  |  |
| Other Backwards Caste | 26.02% (516) | (b) | 0.253 | 19.17% (840) | (b) | 0.283 |
| Scheduled Caste/ Scheduled Tribe | 29.93% (237) | 1.21 (0.96, 1.52) |  | 21.72% (335) | 1.16 (0.94, 1.44) |  |
| General | 26.61% (169) | 1.03 (0.80, 1.32) |  | 21% (342) | 1.12 (0.9, 1.39) |  |
| **Household wealth index quintile** |  |  |  |  |  |  |
| 1 | 22.8% (63) | 0.8 (0.57, 1.13) | 0.1047 | 18.6% (132) | 0.76 (0.58, 1) | 0.015 |
| 2 | 25.6% (134) | 0.94 (0.68, 1.29) |  | 16.1% (171) | 0.64 (0.49, 0.83) |  |
| 3 | 31.7% (202) | 1.27 (0.96, 1.68) |  | 20.6% (275) | 0.86 (0.67, 1.11) |  |
| 4 | 26% (267) | 0.96 (0.74, 1.23) |  | 20.4% (425) | 0.85 (0.67, 1.08) |  |
| 5 | 26.7% (256) | (b) |  | 23% (514) | (b) |  |
| **Currently in school** |  |  |  |  |  |  |
| Yes | 27.1% (664) | 0.99 (0.80, 1.22) | 0.95 | 19.7% (954) | 0.93 (0.78, 1.11) | 0.474 |
| No | 27.3% (258) | (b) |  | 20.7% (563) | (b) |  |
| **Highest level of education attained** |  |  |  |  |  |  |
| None | 22.1% (35) | 0.71 (0.44, 1.15) | 0.119 | 20.4% (112) | (b) | 0.533 |
| 1-7 years | 23.1% (147) | 0.75 (0.55, 1.02) |  | 18.4% (267) | 0.87 (0.59, 1.3) |  |
| 8+ years | 28.4% (740) | (b) |  | 20.5% (1138) | 1 (0.71, 1.41) |  |
| **Mother's education** |  |  |  |  |  |  |
| No education | 26.7% (597) | (b) | 0.146 | 20.2% (1029) | (b) | 0.924 |
| 1-7 years | 32.6% (131) | 1.32 (0.98, 1.78) |  | 20.5% (179) | 1.02 (0.77, 1.35) |  |
| 8+ years | 25.7% (194) | 0.94 (0.74, 1.20) |  | 19.7% (309) | 0.96 (0.78, 1.2) |  |
| **Done paid work in last 12 months** |  |  |  |  |  |  |
| Yes | 30.7% (344) | 1.31 (1.03, 1.66) | 0.027 | 23.6% (353) | 1.3 (1.07, 1.59) | 0.008 |
| No | 25.3% (578) | (b) |  | 19.1% (1164) | (b) |  |
| **Decision-making at household level** |  |  |  |  |  |  |
| Takes decisions independently/jointly | 24.3% (344) | 0.77 (0.62, 0.94) | 0.013 | 20.4% (1144) | 0.91 (0.74, 1.12) | 0.389 |
| No role in decision-making | 29.4% (578) | (b) |  | 19% (373) | (b) |  |
| **Mobility** |  |  |  |  |  |  |
| None | 25.9% (29) | (b) | 0.811 | 18.5% (787) | (b) | 0.004 |
| Two or more | 27.2% (893) | 1.06 (0.61, 1.85) |  | 22.6% (730) | 1.28 (1.08, 1.53) |  |
| **Have any savings** |  |  |  |  |  |  |
| Yes | 29.7% (499) | 1.27 (1.03, 1.56) | 0.020 | 21.4% (765) | 1.15 (0.98, 1.36) | 0.079 |
| No | 24.9% (423) | (b) |  | 19% (752) | (b) |  |
| **Frequent exposure to mass media** |  |  |  |  |  |  |
| Yes | 28.3% (837) | 1.56 (1.12, 2.18) | 0.008 | 20.4% (1082) | 1.04 (0.87, 1.24) | 0.624 |
| No | 20.1% (85) | (b) |  | 19.7% (435) | (b) |  |
| **Peer network** |  |  |  |  |  |  |
| None | 14.5% (13) | (b) | <0.001 | 23% (77) | (b) | 0.727 |
| 1 to 4 friends | 24.8% (512) | 1.94 (0.91, 4.13) |  | 20% (1041) | 0.83 (0.53, 1.3) |  |
| 5 or more friends | 32.5% (397) | 2.84 (1.34, 6.03) |  | 20% (399) | 0.83 (0.53, 1.31) |  |
| **Self-efficacy** |  |  |  |  |  |  |
| Express opinion in at least one of the cases | 25.75% (473) | 0.85 (0.70, 1.04) | 0.117 | 20.48% (773) | 1.04 (0.86, 1.25) | 0.662 |
| Express opinion in neither situation | 28.84% (449) | (b) |  | 19.82% (744) | (b) |  |
| **Ever witnessed domestic violence** |  |  |  |  |  |  |
| Yes | 33.5% (180) | 1.51 (1.19, 1.90) | 0.001 | 27.4% (506) | 1.75 (1.45, 2.12) | <0.001 |
| No | 25% (643) | (b) |  | 17.7% (908) | (b) |  |
| **Discussed SRH issues with parents** |  |  |  |  |  |  |
| Yes | 46.5% (89) | 2.52 (1.74, 3.65) | <0.001 | 23.2% (327) | 1.08 (0.91, 1.28) | 0.374 |
| No | 25.5% (784) | (b) |  | 19.3% (1190) | (b) |  |
| **Aware of at least one STI** |  |  |  |  |  |  |
| Yes | 37.7% (133) | 1.72 (1.26, 2.36) | 0.001 | 25% (187) | 1.36 (1.06, 1.75) | 0.014 |
| No | 26% (789) | (b) |  | 19.6% (1330) | (b) |  |
| **Received any service/ information on STIs/ RTIs** |  |  |  |  |  |  |
| Yes | 27.9% (146) | 1.04 (0.77, 1.40) | 0.775 | 23.2% (327) | 1.26 (1.01, 1.57) | 0.039 |
| No | 27% (776) | (b) |  | 19.3% (1190) | (b) |  |
| **Patient Health Questionnaire-9 categories** |  |  |  |  |  |  |
| Minimal depression | 24.5% (755) | (b) | <0.001 | 16.8% (1014) | (b) | <0.001 |
| Mild depression | 50.3% (135) | 3.10 (2.24, 4.28) |  | 35% (336) | 2.66 (2.13, 3.31) |  |
| Moderate depression | 55.2% (25) | 3.78 (1.79, 7.97) |  | 39.8% (108) | 3.26 (2.35, 4.53) |  |
| Moderately severe depression | 55.1% (6) | 3.77 (0.98, 14.37) |  | 34.8% (37) | 2.64 (1.49, 4.68) |  |
| Severe depression | 100% (1) | - |  | 45.6% (22) | 4.15 (1.56, 11) |  |
| **Use sanitary napkins** |  |  |  |  |  |  |
| Yes | - |  |  | 21.82% (1003) | 1.26 (1.05, 1.52) | 0.011 |
| No |  |  |  | 18.03% (514) | (b) |  |
| **Ever had premarital sex** |  |  |  |  |  |  |
| Yes | 45.7% (136) | 2.51 (1.81, 3.48) | <0.001 | 36.9% (63) | 2.39 (1.62, 3.52) | <0.001 |
| No | 25.1% (786) | (b) |  | 19.7% (1454) | (b) |  |

| **Appendix Table 3: Factors associated with Treatment seeking among male and female adolescents, unadjusted OR (95% CI)** | | | | | | |
| --- | --- | --- | --- | --- | --- | --- |
| **Independent Variable** | **Boys  (N=611)** | | | **Girls  (N=397)** | | |
|  | **% (n)** | **Unadjusted OR  (95% CI)** | **p value** | **% (n)** | **Unadjusted OR  (95% CI)** | **p value** |
| **Location** |  |  |  |  |  |  |
| Rural | 66.2% (343) | (b) | 0.474 | 30.7% (222) | (b) | 0.007 |
| Urban | 69.4% (268) | 1.15 (0.77, 1.72) |  | 23.1% (175) | 1.47 (1.11, 1.94) |  |
| **State** |  |  |  |  |  |  |
| Bihar | 64.3% (250) | 0.86 (0.58, 1.26) | 0.446 | 19.6% (130) | 0.69 (0.46, 1.02) | 0.067 |
| Uttar Pradesh | 67.6% (361) | (b) |  | 25.9% (267) | (b) |  |
| **Religion** |  |  |  |  |  |  |
| Hindu | 67.2% (496) | (b) | 0.577 | 22.2% (247) | (b) | 0.030 |
| Muslim | 64.2% (114) | 0.87 (0.54, 1.4) |  | 31.1% (150) | 1.58 (1.04, 2.38) |  |
| **Caste** |  |  |  |  |  |  |
| Other Backwards Caste | 68.96% (354) | (b) | 0.482 | 27.54% (235) | (b) | <0.001 |
| Scheduled Caste/ Scheduled Tribe | 63.78% (149) | 0.79 (0.53, 1.16) |  | 14.73% (70) | 0.45 (0.3, 0.67) |  |
| General | 64.97% (108) | 0.83 (0.49, 1.41) |  | 27.89% (92) | 1.01 (0.69, 1.48) |  |
| **Household wealth index quintile** |  |  |  |  |  |  |
| 1 | 61.7% (37) | 0.9 (0.43, 1.88) | 0.343 | 14.7% (20) | 0.4 (0.21, 0.73) | 0.027 |
| 2 | 69.2% (90) | 1.27 (0.76, 2.12) |  | 21.4% (43) | 0.63 (0.36, 1.09) |  |
| 3 | 64.3% (134) | 1.01 (0.56, 1.84) |  | 22.3% (63) | 0.66 (0.41, 1.06) |  |
| 4 | 71.9% (182) | 1.44 (0.83, 2.51) |  | 26% (118) | 0.81 (0.53, 1.24) |  |
| 5 | 63.9% (168) | (b) |  | 30.1% (153) | (b) |  |
| **Currently in school** |  |  |  |  |  |  |
| Yes | 70.1% (450) | 1.68 (1.12, 2.52) | 0.012 | 25.1% (258) | 1.09 (0.75, 1.57) | 0.636 |
| No | 58.2% (161) | (b) |  | 23.5% (139) | (b) |  |
| **Highest level of education attained** |  |  |  |  |  |  |
| None | 59.5% (24) | (b) | 0.062 | 20.1% (23) | (b) | 0.094 |
| 1-7 years | 55.7% (91) | 0.85 (0.28, 2.53) |  | 18.2% (54) | 0.88 (0.42, 1.84) |  |
| 8+ years | 69.1% (496) | 1.52 (0.6, 3.86) |  | 26.3% (320) | 1.42 (0.71, 2.83) |  |
| **Mother's education** |  |  |  |  |  |  |
| No education | 68.5% (395) | (b) | 0.103 | 23.5% (254) | (b) | 0.337 |
| 1-7 years | 69% (91) | 1.02 (0.58, 1.79) |  | 23.1% (49) | 0.97 (0.55, 1.72) |  |
| 8+ years | 58% (125) | 0.63 (0.41, 0.97) |  | 29% (94) | 1.33 (0.9, 1.95) |  |
| **Done paid work in last 12 months** |  |  |  |  |  |  |
| Yes | 64% (231) | 0.81 (0.54, 1.22) | 0.338 | 23.8% (293) | (b) | 0.466 |
| No | 68.5% (380) | (b) |  | 26.4% (104) | 1.14 (0.79, 1.66) |  |
| **Decision-making at household level** |  |  |  |  |  |  |
| No role in decision making | 68.6% (210) | (b) | 0.223 | 22.6% (289) | (b) | 0.042 |
| Takes decisions independently/jointly | 63.7% (401) | 0.8 (0.56, 1.14) |  | 31.1% (108) | 1.55 (1.01, 2.36) |  |
| **Mobility** |  |  |  |  |  |  |
| None | 67.4% (19) | (b) | 0.932 | 22.4% (184) | (b) | 0.137 |
| Two or more | 66.7% (592) | 0.96 (0.44, 2.09) |  | 27% (213) | 1.27 (0.92, 1.76) |  |
| **Have any savings** |  |  |  |  |  |  |
| Yes | 67% (324) | 1.03 (0.71, 1.48) | 0.859 | 26.5% (216) | 1.23 (0.88, 1.73) | 0.214 |
| No | 66.3% (287) | (b) |  | 22.5% (181) | (b) |  |
| **Frequent exposure to mass media** |  |  |  |  |  |  |
| Yes | 67.2% (558) | 1.21 (0.71, 2.06) | 0.463 | 29.5% (314) | 2.18 (1.57, 3.03) | <0.001 |
| No | 62.7% (53) | (b) |  | 16% (83) | (b) |  |
| **Peer network** |  |  |  |  |  |  |
| None | 16.6% (5) | (b) | 0.001 | 19.1% (14) | (b) | 0.686 |
| 1 to 4 friends | 70.5% (349) | 11.97 (2.83, 50.7) |  | 24.9% (282) | 1.4 (0.64, 3.03) |  |
| 5 or more friends | 63.3% (257) | 8.64 (2.05, 36.37) |  | 24.5% (101) | 1.37 (0.63, 2.96) |  |
| **Self-efficacy** |  |  |  |  |  |  |
| Express opinion in one or both cases | 68.88% (324) | 1.21 (0.83, 1.77) | 0.296 | 26.97% (233) | 1.32 (0.95, 1.82) | 0.091 |
| Express opinion in neither situation | 64.48% (287) | (b) |  | 21.84% (164) | (b) |  |
| **Ever witnessed domestic violence** |  |  |  |  |  |  |
| Yes | 65.3% (120) | 0.83 (0.52, 1.34) | 0.465 | 20.3% (116) | 0.68 (0.47, 0.99) | 0.046 |
| No | 69.1% (436) | (b) |  | 27.1% (263) | (b) |  |
| **Discussed SRH issues with parents** |  |  |  |  |  |  |
| Yes | 79.7% (64) | 2.06 (1.04, 4.08) | 0.037 | 28.2% (307) | 2 (1.39, 2.88) | <0.001 |
| No | 65.6% (516) | (b) |  | 16.3% (76) | (b) |  |
| **Aware of at least one STI** |  |  |  |  |  |  |
| Yes | 74.6% (93) | 1.55 (0.95, 2.54) | 0.078 | 33.7% (66) | 1.68 (1.07, 2.63) | 0.023 |
| No | 65.4% (518) | (b) |  | 23.1% (331) | (b) |  |
| **Received any service/ information on STIs/ RTIs** |  |  |  |  |  |  |
| Yes | 64.5% (98) | 0.9 (0.54, 1.5) | 0.709 | 24.1% (87) | 0.97 (0.6, 1.57) | 0.911 |
| No | 67% (513) | (b) |  | 24.6% (310) | (b) |  |
| **Ever had premarital sex** |  |  |  |  |  |  |
| Yes | 67.7% (90) | 1.05 (0.63, 1.75) | 0.832 | 13.6% (13) | 0.47 (0.22, 0.96) | 0.039 |
| No | 66.5% (521) | (b) |  | 25% (384) | (b) |  |
